# Exploring the Importance of Gender, Family Affluence, Parenting Style and Loneliness in Cyberbullying Victimization and Aggression among Romanian Adolescents

**DOI:** 10.3390/bs12110457

**Published:** 2022-11-17

**Authors:** Magdalena Iorga, Lavinia Maria Pop, Irina Croitoru, Elena Hanganu, Dana-Teodora Anton-Păduraru

**Affiliations:** 1 Behavioral Sciences Department, “Grigore T. Popa” University of Medicine and Pharmacy, 700115 Iasi, Romania; 2 Faculty of Psychology and Education Sciences, “Alexandru Ioan Cuza” University of Iasi, 700554 Iasi, Romania; 3 Preventive Medicine and Interdisciplinarity Department, “Grigore T. Popa” University of Medicine and Pharmacy, 700115 Iasi, Romania; 4 Pediatric Department, “Grigore T. Popa” University of Medicine and Pharmacy, 700115 Iasi, Romania

**Keywords:** cyberbullying, adolescent, loneliness, victim, perpetrator, bystander, self-esteem, parental style, family affluence, cyberaggression, cybervictimization, school

## Abstract

The increasing phenomenon of cyberbullying among adolescents needs parental, educational, and social intervention. The study aimed to identify the prevalence of cyberbullying among Romanian adolescents and the importance of gender, family-related factors, self-esteem, and parental styles in both victims and perpetrators. A total of 835 adolescents aged 10–19 years were included in the research. An online questionnaire was specially constructed for this research, gathering socio-demographic and family-related data along with information about cyberbullying as a victim, aggressor, or bystander, and strategies used in order to deal with it. Four psychological scales were used to evaluate self-esteem, loneliness, cybervictimization/cyberaggression, and parental style. (3) Results showed that the most common age for a personal smartphone is M = 10.24 ± 2.43. The main reasons why students use these networks are primarily chatting and fun and less for academic tasks. During the week, adolescents spend 5.53 ± 2.75 h on social media, while during weekends, the duration of smartphone usage almost doubles. Girls are the most common victims of cyberbullying, and less than three-quarters of students believe that aggressors can be both girls and boys, and only a quarter of them have reported an incident. Family affluence, the relationship with parents and classmates, the presence of loneliness and sociodemographic factors were found to be in a strong relationship with the presence of aggression and/or victimization among adolescents. Cyberaggression was found to be positively correlated with the aggressive parental style and negatively correlated with the compassionate and avoidant parental styles. Results are crucial for identifying cyberbullying actors and preventing the negative effects of cyberbullying on psychological, social, and academic life for students, parents, and teachers.

## 1. Introduction

The rapid advances in Information and Communication Technologies (ICT) have changed the way people interact and communicate with each other, contributing to an extremely high amount of transmitted content, the speedy exchange of ideas, a large quantity of information, and connecting people from all over the world. The positive aspects of using technologies in daily communication caused negative consequences that cannot be ignored: the rapid spread of fake information, increased time using social networks, promotion of a false image, easy way to practice aggressive behaviors and difficulties determining the identity of the sender.

Data provided by UNICEF showed that almost 98% of the population use smartphones in their daily lives [1]. One in three internet users globally is a child and usually owns a smartphone at the age of 10. The number of hours spent on the internet increases with age, along with all the associated risks. In the last decade, scientific literature continuously provided a lot of information about the consequences of the increasing use of the internet among children and young people. The more frequent use of mobile phones or the internet was associated with depression [2], self-isolation [3], anxiety [4], poor body image [5,6], and aggressive behaviors [7].

Cyberbullying (CB) has been defined as aggressive and deliberate behavior that is frequently repeated over time, carried out by a group or an individual using electronics and aimed at a victim who cannot defend himself/herself [8]. Patchin and Hinduja defined cyberbullying as being more frequent than traditional bullying and described it as deliberate and repeated harm done with technology [9,10]. Unlike adults, who can act when they are bullied online, children do not have the necessary tools to report or defend themselves. The Social Defeat Model showed that the loser of a fight among animals of the same species may show such signs as increased sleep, lowered testosterone, and less exploratory behavior [11]. In the case of young people, cyberbullying can be followed by poor academic results, absenteeism, and self and auto-aggression. Because bullying and cyberbullying are repetitive behaviors, dealing with these problems needs the presence of an adult who must act (parent, teacher, school principal, etc.). If the adult does not interfere, the victim will try to solve the problem by herself/himself, the most frequent behavior being an offensive one in order to discourage the aggressor. In its turn, the victim will become an aggressor. That is why cyberbullying has become a systemic public health issue [11,12] with a great impact on the physical, psychological, and social health of all those involved.

Recent studies have revealed an alarming increase in internet use and cyberbullying among adolescents in developed countries [13,14]. Victims and bystanders of bullying experience anxiety, poor self-esteem [15], and depressive symptoms [16] as well as frequent somatic complaints [17] suicides [18,19] and homicides [20]. Being bullied at school may be a contributing factor to the development of all mentioned consequences in adulthood. The cyberbullying experienced in adolescence is a predictor of bullying behaviors in social relations and professional lives as adults [21].

If traditional bullying develops in real settings with a real aggressor and with real witnesses, in cyberbullying, the known or unknown aggressor can follow the victim everywhere, every time, and the number of witnesses can be considerably high. That is why the consequences of cyberbullying are more severe than in the case of traditional bullying, one of the most important being self-aggressive behaviors. For example, suicide attempts were found to be a great risk in cyberbullying experiences among adolescents. A study conducted by Campisi et al., in 2019, among adolescents from ninety countries revealed that significant indicators associated with suicidal ideation or attempt included being bullied, having no close friends, being in a physical fight, and/or having had a serious injury [11].

The “EU Kids Online” study of over 25,000 children between the ages of 9 and 16 reported a prevalence of cybervictimization ranging between 2 and 14%. The highest rates were found in Estonia and Romania, and the lowest in Italy and Portugal [22]. In 2018, a study conducted by Athanasiou et al. [23] among 14–17-year-old adolescents across seven European countries, showed that the highest rate of cyber victimization was found in Romania (37.3%), and the lowest in Spain (13.3%). A previous study conducted by Grigore & Maftei [2] on 501 middle adolescents aged 12 to 15 years showed no significant associations between the participants’ status, gender, or age, but participants with higher levels of state anxiety and depression were more likely to be victims. In another study, Cucos et al. [24] identified significant negative associations between intercultural sensitivity and cyber-perpetration, cybervictimization, and cyber-bystander behavior among 241 high school students, while Turliuc et al. [25] identified the relationship between cyberaggression and depressive symptoms in research including 310 adolescents. 

Data about cyberbullying as an increasing phenomenon in Romania is scarce. The aim of the present research was to identify the prevalence of cybervictimization and cyberaggression among adolescents, and to highlight the relationships between gender, family affluence, parenting styles, and loneliness. The World Health Organization defines an adolescent as a person aged 10–19, so, for the present study, only this category was considered. 

## 2. Materials and Methods

### 2.1. Study Design and Study Population

The study was carried out within the project TECPC—Together Everyone Can Prevent Cyberbullying (2020-1-RO01-KA226-SCH-095269), in the framework of the Erasmus+ Programme—KA2 Strategic Partnerships Digital Education Readiness. 

The study is a cross-sectional, school-based survey conducted between May-June 2022. Data collection was performed using a snowball sampling methodology focusing on the general student population from different departments from North-Eastern Romania, in rural and urban areas. Schools were informed about the objectives of the research and the questionnaires were delivered after the approval of the principals was obtained. The questionnaire was distributed online. Target participants were adolescents from secondary/gymnasium and high schools/colleges (in Romania, the mentioned study levels include students aged 10–14 and 15–19 years old). Participants were informed about the purpose of the study and the confidentiality of data, and they were informed that they could withdraw from the study whenever they wanted, without consequences. No incentives were given to participants. 

The inclusion criteria were questionnaires filled in by children enrolled in private or public school, aged 10–19 years old, submitting fully filled in questionnaires. The criteria for excluding questionnaires from the research were questionnaires not fully completed and questionnaires submitted after the deadline. A total of 835 questionnaires were finally included in the research.

### 2.2. Study Instruments

The questionnaire was constructed using the Google Forms application (Alphabet, Mountain View, CA, USA) and was developed in order to address the prevention, recognition, and intervention of online harassment among adolescents. 

The questionnaire was built following solid research of specialized literature on cyberbullying as well as discussions with teachers, school principals and students of various ages.

(a) The first part of the questionnaire gathered socio-demographic information (like age, gender, level of education of children and their parents, home environment, school environment, and members of the household).
  Data about family income was measured using the *Family Affluence Scale* (FAS) which was developed first in Scotland as a measure of family affluence. It was proved that, at a young age, children did not have accurate information on their family’s finances, and adolescents too were not informed about the family income. Therefore, this evaluation was found to be a less intrusive and more comprehensible approach that had to be applied in order to evaluate socioeconomic status among children and teenagers [26].
  The Family Affluence Scale (FAS), a four-item measure of family wealth, was developed in the WHO Health Behaviour in School-aged Children Study as an alternative measure, and in 2001–2002 the scale was composed of four items:
  1. Does your family own a car, van or truck? (No [0]; Yes, one [1]; Yes, two or more),
  2. Do you have your own bedroom for yourself? (No [0]; Yes [1]),
  3. During the past 12 months, how many times did you travel away on holiday with your family? (Not at all [0]; Once [1]; Twice [2]; More than twice [3]),
  4. How many computers do your family own? (None [0]; One [1]; Two [2]; More than two [3]).
(b) The second part includes questions about children’s satisfaction with the relationships with parents, classmates, colleagues from school, friends, and teachers. Self-assessed items were constructed, and responses were assessed on a 5-point Likert scale. Other questions in this section include items about grades obtained in the previous year, as well as the relationship between mother and father from the children’s perspective, children’s relationships with parents, the main decision-maker in the family, self-assessment of social position (leader, popular, or lonely person), positioning in the school learning situation compared to classmates, number of best friends, and number of children in the class.
(c) The third part targeted bullying and cyberbullying behaviors, and included items that refer to children’s views on the gender of people who are most often abusers or abuse others (boys or girls); if they have ever been an online abuser or a victim of physical or online bullying; if they have colleagues who terrorize others; if they have seen colleagues who are terrorized physically or online; and if they have reported the incident in those cases.
(d) The fourth part of the survey collected information about the use of mobile phones and the internet; the main reason for using the internet; the time spent on average on a usual working day and on a weekend day on the internet; the age at which children received their first phone call; how often they socialize with people they know on the internet; as well as their parents’ behavior towards them regarding excessive phone use (blaming, insulting, restricting access).
(e) The final part of the questionnaire addressed several standardized scales, presented above:
  - *Rosenberg self-esteem scale* consists of 10 items and it is a self-report instrument for evaluating individual self-esteem (Rosenberg, 1965). RSES is scored using four response choices, ranging from strongly agree to strongly disagree [27].
  - *UCLA Loneliness scale* (ULS-8) contains the 20 items selected from the third revised version UCLA Loneliness Scale of Russell et al., in 1980. This instrument is scored on a 4-point Likert scale with values ranging from 1 (never) to 4 (always). The UCLA is a commonly used tool developed to measure one’s subjective feelings of loneliness as well as feelings of social isolation [28].
  - *The Cyber-aggression Scale* (CYB-AGS) constructed by Buelga & Pon in 2012 comprised 18 items rated on a 5-point Likert-type scale ranging from 1 (never) to 5 (always). These items measure the adolescent’s experience as a cyberbullying perpetrator (directly or indirectly) in the past 12 months [29].
  - *The Cyber victimization Questionnaire* (CYVIC) is a self-report instrument composed of 19 items, each one of which presents an aggression suffered through mobile phone or the Internet constructed by Álvarez-García et al. in 2017. The students should mark the frequency with which they were the victim of each one of these situations in the past three months, on a 4-point Likert-type scale [30].
  - *The Parenting Styles and Dimensions questionnaire* (PSDQ) constructed by Batool, in 2016 has 40 items and it was designed to measure parenting styles grouping them in six typologies of supportive, controlling, compassionate, aggressive, avoidant, and orthodox [31].

### 2.3. Statistical Analysis

All analyses for this research were performed using IBM Statistical Package for Social Sciences (SPSS) Statistics for Windows, version 26 (SPSS Inc., Chicago, IL, USA). Results for descriptive statistics were expressed as means and standard deviations (SD). 

The normality of data distribution was tested by using the Kolmogorov-Smirnoff test. Given the fact that all data points are not normally distributed, a bivariate analysis will be performed, and non-parametric tests will be applied. 

The Mann–Whitney test was performed to assess comparative results considering gender, living environment, level of education, and school environment. The Kruskal–Wallis H test was used to determine if there were statistically significant differences between two groups of an independent variable on a continuous or ordinal dependent variable, considering family affluence, mothers’ level of education, and fathers’ level of education.

The Spearman correlation was used to test the relationship between variables. A *p*-value < 0.05 was considered statistically significant. 

### 2.4. Ethical Statement

The study was conducted in accordance with the Declaration of Helsinki and approved by the Institutional Review Board No 192/30.04.2022 of EuroEd School, Iași, Romania.

## 3. Results

### 3.1. Socio-Demographic Data, Family Characteristic, Financial Status

Sociodemographic data family-related data, financial status, environmental characteristics, and household-related information were gathered.

The participants were middle school (24.8%, n = 207) and high school (75.2%, n = 628) students, and aged M = 15.84 ± 1.82 (with a minimum of 10 and a maximum of 19 years old).

Most students were female (63.0%, n = 526) and more than half of them came from rural areas (58.3%, n = 487). However, the majority stated that their school is in a city (92.6%, n = 773), and more than half of the participants stated that their school was not in the same locality as their home (52.2%, n = 436).

Children and adolescents were asked if they had at least one parent working abroad, and we identified that 1/4 of students had at least one parent working in another country (n = 205, 24.5%). Most students lived with both parents (72.6%, n = 606), few lived with only one parent—mother (13.2%, n = 110) or father (3.5%, n = 29), with grandparents and/or other relatives (10.8%, n = 90), and 18.3% (n = 153) had parents that were not living together.

In terms of parents’ level of education, the analysis of responses revealed that almost half of mothers and fathers graduated high school, and a quarter of them had a higher level of education. The study also collected data about the number of children in the family. The analysis of the data showed that 18.0% (n = 150) were only children, 50.5% (n = 422) had a brother or a sister, 17.8% (n = 149) had two siblings, and 13.7% (n = 114) stated that they had more than 3 brothers or sisters. The number of children in the family was M = 2.33 ± 1.04. More details about the parents’ level of education and the number of children in the family are presented in Table 1.

Family financial status was measured using a 4-item scale, the *Family Affluence Scale (FAS*). A composite FAS score was calculated for each student based on the answers to these four items. Thus, the total scores varied between 0 points—which indicates low affluence (2.6%, n = 22), and 9 points (7.2%, n = 60)—which indicates high affluence. Scores between 3 and 5 points indicate medium affluence (37.2%, n = 310), the results showing that approximately half of the participants (49.2%, n = 411) had high family affluence. The average score was M = 5.30 ± 2.33. 

### 3.2. Relationship with Family, Friends and Colleagues

Most students considered that the relationship between their parents was collaborative (n = 677, 81.1%) and a small number of participants declared that this relationship was conflictual (n = 62, 7.4%) or that there was no relationship between their parents (n = 96, 11.5%). Similarly, more than half of the participants considered that their parents had a normal relationship (68.6%, n = 573), but there were also cases in which disputes still occurred—sometimes (14.4%, n = 120) or quite frequently (4.7%, n = 39)—or even cases in which the parents had no relationship and did not talk to each other (12.3%, n = 103)

Students were also asked how they appreciated the relationship between their parents. On a scale of 1 (very dissatisfied) to 5 (very satisfied), about half of the students said that they were very satisfied with the relationship between their parents (48.9%, n = 408), while the rest evaluated it as being very unsatisfactory (2.5%, n = 21), unsatisfactory (6.3%, n = 53), neither satisfactory nor unsatisfactory (18.0%, n = 150) to satisfactory (24.3%, n = 203). 

More than half of the children declared that there was not a single family member who made decisions, but that there was a shared decision between the members of the family (64.7%, n = 540). 

The relationship between children and parents was considered to be quite peaceful usually, more than half of the students stated that their mothers (67.3%, n = 562) and fathers (68.5%, n = 572) did not offend them and never shouted at them, and the cases in which mothers (0.4%, n = 3) or fathers (0.6%, n = 5) always screamed at children were quite scarce.

Students were asked to express their satisfaction with their relationship with different categories of people on a scale of 1 (very dissatisfied) to 5 (very satisfied). In general, students were most satisfied with their relationships with friends and parents. Students stated that they had between none (7.8%, n = 65) and more than five best friends (12.7%, n = 106), with an average of M = 2.36 ± 0.80. Regarding the number of children in the class, the students stated an average of M = 27.88 ± 3.67. More self-rated items are described in Table 2. 

### 3.3. Self-Evaluation of the Academic Results and Self-Positioning within the Group 

On a Likert-like scale from 1 (very unsatisfied) to 5 (very satisfied), the analysis of answers showed that 1/3 of students were either satisfied with their grades (36.2%, n = 302) or indifferent (30.3%, n = 253), the average being M = 3.59 ± 1.01. In addition, more than half of the students reported achieving medium learning results compared to their classmates (59.0%, N = 493). 

Additionally, using the same scale, students were asked to mention their degree of agreement/disagreement that they could be a popular person, a group leader, or, conversely, a lonely person. Thus, the results showed that students evaluated themselves as being more solitary than popular people, and fewer considered themselves to be leaders of their group. Detailed results are presented in Table 3.

### 3.4. Use of Internet and Mobile Phones

The results indicated that the average age at which children had their first personal phone is M = 10.24 ± 2.43. The Mann–Whitney test (U = 70,413.00, Z = −4.203, *p =* 0.001) revealed that there was a significant difference at this item when considering the living environment, with children living in a city (Mdn = 10.00) receiving the first smartphone at a younger age than children living in a village (Mdn = 11).

The main reasons why students used these networks were primarily chatting (55.1%, n = 460) and for fun (35.7%, n = 298), and less so for help in solving academic tasks (9.2%, n = 77). On average, students spent 5.53 ± 2.75 h on social media during the week, while at the weekends they spent, on average, 10.82 ± 9.56. 

On a scale from 1 (never) to 5 (often), students were asked how often they talked on the internet with people they knew. More than half of the students spoke at least once to an unknown person, with only 15% (n = 125) of the students being those who never spoke to someone unknown. The results indicate an average of M = 2.96 ± 1.26. However, most students (87.5%, n = 731) had blocked someone on social media at least once.

Moreover, students were asked if their parents blamed them for using their phones too much. More than half of the students admitted that their parents did this sometimes (60.2%, n = 503), while others stated that their parents never blamed their children (17.5%, n = 146) or, on the contrary, did it frequently (13.2%, n = 110), or all the time (9.1%, n = 76). However, most parents did not take action because their children spent too much time on the phone, with 73.1% (n = 610) never restricting access to the phone, and 23.0% (n = 192) doing so only occasionally.

### 3.5. Bullying and Cyberbullying Behaviors

Three-quarters of the students in the study (72.9%, n = 609) stated that they did not have classmates who cyberbullied others. Furthermore, in about three-quarters of the cases, students considered that girls were the most common victims of cyberbullying (73.2%, n = 611). In addition, less than three-quarters of the students (71.1%, n = 594) believed that aggressors could be both girls and boys alike. 

Most students said they had never been victims of cyberbullying (81.2%, n = 678), and that they had never cyberbullied other children (86.7%, n = 724).

In terms of reporting an online bullying incident in the classroom or school, less than half of the students (38.3% n = 320) stated that they had not seen other children being bullied online in the classroom or school, and 42.0% of children did not report any incidents of a kid being bullied online (messages, social media, chatrooms) because they had not seen any messages. Detailed results are presented in Table 4. 

### 3.6. Global Self-Esteem, Loneliness, Cyberbaggression, Cybervictimization and Parental Style Scales

- *Global self-esteem*

Global self-esteem scores as measured with the Rosenberg self-esteem scale (RSES) ranged from 17 up to 30 (M = 24.02 ± 1.81). Most students had a normal self-esteem (82.4%, n = 688). For the present study, Cronbach Alpha score was 0.816.

- *Loneliness*

The total score for Loneliness scale was on average M = 38.98 ± 9.88, scores ranging from 20 (0.4%, n = 3) to 71 (0.1%, n = 1). More than half of the students (54.9%, n = 458) had a moderate level of loneliness and more than a quarter had a high level of loneliness (33.3%, n = 278). Cronbach’s alpha of 0.882 showed that the scale had a high level of internal consistency.

- *Cyberaggression*

The Cronbach Alpha score for Cyber-aggression was 0.911 for the total scale. For subscales, we obtained the following results: *Indirect Cyber Aggression*—M = 10.28 ± 3.65, Cronbach Alpha = 0.830, and *Direct Cyber Aggression*—M = 10.90 ± 3.02, Cronbach Alpha = 0.901.

The results proved that the respondents did not have such a high level of cyber aggression. The total CYB-AGS score was on average M = 21.18 ± 6.19, with scores ranging from 18 (40.7%, n = 340) to 71 (0.1%, n = 1).

- *Cybervictimization*

The total score for Cyber victimization was on average M = 19.84 ± 4.99, with scores ranging from 15 (12.5%, n = 104) to 47 (0.1%, n = 1). The scale had a high level of internal consistency, as determined by a Cronbach’s alpha of 0.879 for the total scale.

- *Parental style*

The total score for the PSDQ scale was M = 119.19 ± 17.43, scores ranging from 39 (0.1%, n = 1) to 190 (0.2%, n = 2). The Cronbach Alpha score was 0.841. 

### 3.7. Loneliness and School Environment

Important comparative results were identified on the Loneliness scale, in terms of the environment in which the students’ schools were located (U = 19,090.50, Z = −2.668, *p* = 0.008), in the sense that students whose school was located in rural areas had a higher score on the loneliness scale (Mdn = 40.50), as opposed to students studying in the city (Mdn = 37.00). Participants were asked if they had classmates who assaulted other children. Thus, significant differences were identified between this variable and the Loneliness scale (U = 59,366.50, Z = −3.053, *p* = 0.002), in the sense that those who had aggressors among their classmates obtained a higher score on the Loneliness scale (Mdn = 40.00), compared to those who did not have such classmates (Mdn = 37.00).

Significant differences were also found on the loneliness scale in terms of family affluence (U = 15,173.50, Z = −5.762, *p =* 0.001), with students with a low family affluence score having a higher loneliness score (Mdn = 45.00) than those with a high family affluence score (Mdn = 35.00).

### 3.8. Cyberbaggression and Cybervictimization in School Environment

There are significant differences on this scale in terms of the school environment (U = 19,991.50, Z = −2.185, *p* = 0.029) in the sense that children studying in a city had a lower score on cyber victimization (Mdn = 18.00) compared to children studying in a village (Mdn = 19.00).

The Mann–Whitney test (U = 1632.50, Z = −3.894, *p* = 0.001) revealed that there were significant differences on the CYVIC scale in terms of participants’ satisfaction with the relationship with their classmates, with children who were very satisfied with the relationship with their classmates having a lower score on this scale (Mdn = 17.50) compared to children who were very dissatisfied with the relationship with their colleagues having a higher score on this scale (Mdn = 20.00). Moreover, similar results were identified on the cyber victimization scale and the relationship with parents (U = 2340.50, Z = −3.536, *p* < 0.001) and teachers (U = 1947.00, Z = −3.026, *p* = 0.002), which means that students who had a very good relationship with parents (Mdn = 18.00) and teachers (Mdn = 17.00) had lower scores on this scale compared to those who were very dissatisfied with the relationship with parents (Mdn = 21.00), versus teachers (Mdn = 20.50).

Significant differences were identified regarding the variable which asked students if they had classmates who bullied other children and the cyber aggression scale (U = 51,515.50, Z = −5.804, *p* < 0.001), in the sense that those who had classmates that bullied others had a higher score on the cyber aggression scale (Mdn = 20.00) compared to those who did not have such classmates (Mdn = 19.00).

A Mann–Whitney test (U = 53,452.00, Z = −7.904, *p* = 0.001) revealed that there were significant differences on the CYB-AGS scale in terms of participants’ responses when confronted with bullying behavior, with children who had seen bullying behavior scoring higher on the cyber aggression scale (Mdn = 20.00) than children who had not seen bullying behavior, who scored lower (Mdn = 18.00). Additionally, the comparative analysis (U = 44,705.50, Z = −10.305, *p* < 0.001) showed that children who had seen other kids being bullied had a higher score on the cyber victimization scale (Mdn = 19.00) than children who had not seen such behaviors (Mdn = 17.00). Moreover, the comparative analysis showed a significant difference in terms of reporting bullying events to an adult and the cyber aggression scale (U = 28,788.00, Z = −2.408, *p* = 0.016) in the sense that students who reported these events had a lower score on CYB scales (Mdn = 19.00) compared to those who did not report bullying behaviors (Mdn = 20.00).

### 3.9. Victim and Aggressor Roles—A Vicious Cercle

The results showed that there was a negative correlation between the CYB-AGS and children’s satisfaction with their relationship with their friends (r = −0.125 **, *p* < 0.001), in the sense that the more dissatisfied the children were with this relationship, the more likely they were to become cyber aggressors. 

A strong positive correlation was identified between the total score of CYB-AGS and the item that refers to the frequency with which children were victims of cyberbullying. Thus, the results (r = 0.486 **, *p* < 0.001) showed that the more often it happened that children were the victims of cyberbullying, the higher the score on the CYB-AGS scale. 

The relationship between parents (r = 0.143 **, *p* < 0.001) and the frequency with which children happened to be victims of cyberbullying (r = 0.301 **, *p* < 0.001) were positively correlated with the scores obtained on the CYVIC scale, in the sense that the more conflictive the parents’ relationship was and the more often the children were terrorized online, the greater the aggression suffered by children through the Internet. 

Family affluence is negatively correlated with the CYVIC scale (r = −0.068 *, *p* = 0.049) in the sense that the higher the family affluence, the lower the score on the cyber victimization scale. 

A negative correlation was also identified between the CYVIC score and students’ satisfaction with their relationship with friends (r = −0.214 **, *p* < 0.001), meaning that the less satisfied they were with their friends’ relationship, the more likely they were to become victims of cyberbullying. 

A positive correlation was also identified between the responses to the item on how often children bullied others while online and the CYVIC scales (r = 0.301 **, *p* < 0.001), in the sense that the higher the level of cyber aggression was, the higher the scores of children on this scale.

The analysis of data showed that the higher the level of loneliness, the higher the scores on these subscales related to cyber aggression, direct or indirect, impersonation, online exclusion, visual-sexual, and written-verbal cyber victimization.

The study found that some of the parenting styles were negatively correlated with the results obtained in these subscales, which means that different types of parents predisposed children to obtain higher overall scores of cyber aggression and cyber victimization. Details regarding these significant correlations are presented in Table 5.

### 3.10. The Imfluence of the Parenting Styles 

The Mann–Whitney test (U = 56,161.50, Z = −2.937, *p* = 0.003) revealed that there were significant differences in the PSDQ scale between children’s educational levels, with middle school students scoring higher (Mdn = 120.00) than high school students.

The Mann–Whitney test (U = 848.00, Z = −6.203, *p* = 0.001) showed that there were significant differences on the PSDQ scale in terms of participants’ satisfaction with the relationship with their parents, with children who were very satisfied with the relationship with their parents having a higher score at this scale (Mdn = 125.00) compared to children who were very dissatisfied with the relationship with their parents having a lower score at this scale (Mdn = 100.00).

A significant difference in terms of reporting bullying events to an adult and the PSDQ scale was revealed (U = 28,561.50, Z = −2,495, *p* = 0.013) in the sense that students who reported those events had a higher score on the PSDQ scale (Mdn = 123.00), compared to those who did not report bullying behaviors (Mdn = 118.00).

The analysis of the data showed that the mother’s level of education was positively correlated only with supportive parenting styles, in the sense that the higher the mother’s level of education, the more likely they were to become a supportive parent. In contrast, the relationship between parents evaluated from the children’s perspective was negatively correlated with almost all parenting styles, while family affluence was negatively correlated only with aggressive parenting styles and positively correlated with supportive, compassionate, avoidant, and controlling parenting styles.

In addition, the results showed that supportive parenting styles were negatively correlated with cyber victimization scores in the sense that the better the parents fitted into this parenting style, the lower the level of cyber victimization. 

The scores on the cyber aggression scale were positively correlated with the aggressive parental style and negatively correlated with the compassionate and avoidant parental styles, in the sense that the better the parents fitted into the aggressive parenting typology, the higher the level of cyber aggression. The better the parents fitted into the compassionate and avoidant parenting styles, the lower the level of cyber aggression. Details are presented in Table 6.

## 4. Discussion

The study included respondents aged 10–19, enrolled in private and public schools, from both rural and urban areas. The analysis of data showed that a quarter of the students had at least one parent working abroad. 

The analysis of the answers showed that students owned a personal smartphone around the age of 10, and students living in urban areas usually had a phone at a younger age compared to respondents from rural areas. The primary reasons for using smartphones were chatting and entertainment, rather than academic tasks. During the week, adolescents spent 5.53 ± 2.75 h on social media, while at the weekends, their smartphone usage nearly doubled (10.82 ± 9.56) Almost 90% of subjects said that they blocked people who behaved aggressively online (87.5%).

We identified that students believed that girls were more prone to being victims of cyberbullying than boys, and that the aggressor could be either a girl or a boy. The literature presents different statistics regarding the prevalence of cyberbullying considering gender that seems to be a predictor of cyber-bystanding in some countries but not in others. Girls are significantly more likely than boys to be cyber-bystanders in Australia [32], the Islamic Republic of Iran [33], and Spain [34], and among Jewish adolescents in Israel [19]. However, there is no difference by gender among Arab adolescents in Israel [19] and in the Republic of Korea [35,36].

Most of the subjects considered the relationship between their parents to be collaborative (81.1%). In general, respondents declared that they were more satisfied with the relationship with their parents and friends, and less with teachers and other people in the school. More than half of the students stated that their mothers (67.3%) and fathers (68.5%) did not offend them and never shouted at them. 

According to our findings, more than a quarter of the adolescents believed that the most common adults who learned about the incident were their parents, and less than half of them reported the incident to their teachers or principals. A similar percentage stated that they never reported a bullying incident to someone else. Data from the scientific literature showed that students avoided reporting incidents for different reasons: their previous experience proved that they did not receive the expected support; the victim was threatened; or, due to the presence of witnesses who did not interfere, the victim considered that this was a banned problem, accepted by the group. Corroborating the data, a good relationship with the parents made the adolescents more confident, manage their relationships better, and look for support in case of need. Higher rates of cyberbullying were reported among those whose parents’ educational level was low or middle compared to high [37], and among families where there was a good relationship between children and parents. 

Parental monitoring in relation to cyberbullying was found to be a protective factor for online risks. Regarding the extent of adolescent Internet and SNS use, the study of Haddon et al. [38] showed that higher levels were associated with an increased risk of cybervictimization among adolescents. We identified that the higher the mother’s level of education, the more likely they were to become a supportive parent.

Similar results were reported in other studies. For example, lower levels of victimization and aggression in bullying and cyberbullying were found to be linked to the indulgent democratic or normative democratic styles, and higher levels to the authoritarian and strict styles, in a study conducted by Gómez-Ortiz et al. [39]. In a previous investigation, the same team identified that both the use of psychological control and the lack of supervision by parents were risk factors that increased the likelihood of children bullying their peers or being victimized by them [40]. The authors identified that certain parenting styles facilitated the construction of a relaxed family environment by offering affection, open communication, and encouraging minors’ autonomy. By creating rules and family discipline, the parents avoid coercive practices such as physical punishment or psychological control that seem to increase the risk of aggression [41].

The literature focusing on cyberbullying is more developed regarding empathy, emotional regulation, and psychological distress in perpetrators, victims, and perpetrator-victims of cyberbullying, and not much information presents the profile of the active or passive bystander. We found that adolescents from urban areas obtained a lower score on cybervictimization. A higher score for cybervictimization was also found in subjects with low satisfaction regarding their relationship with their classmates and parents. Moreover, we found that students who had classmates that bullied others, and those who were witnesses to bullying incidents had a higher score on the cyber aggression scale. Adolescents who reported bullying events obtained a lower score on both CYB scales. 

The results showed that adolescents who had aggressors among their classmates obtained a higher score on the Loneliness scale compared to those who did not have such classmates. Additionally, significant differences were identified on the scale of loneliness in terms of family affluence, showing that students whose families had a low family affluence score obtained a higher score. Family affluence was found to be negatively correlated with the CYVIC scale, in the sense that the higher the family affluence, the lower the score on the cyber victimization scale. 

Cyberbullying, the ”silent killer” [42] is widespread across countries [43] with rates ranged from 14.6% to 52.2% (cybervictimization) and 6.3% to 32% (cyberaggression) conforming to Zhu and his team [12].

Younger children were found to frequently report incidents than older students and studies showed that incidents increase with age, but cyberbullying reports decrease with age. Blomqvist et al. [44] found that adolescents may be less likely to disclose victimization compared to youngest ones because of their need for increased autonomy and the desire to solve the problems by themselves.

As age, gender was found to be an important variable. In many studies, more girls reported being victims of cyberbullying compared to boys, but a lot of studies showed that teenage girls are more frequent users of social networks than boys, facilitating cyber incidents [23,45,46]. Additionally, even if statistics showed that boys spend more time on using the internet, it is important to take into consideration the purpose of the use. Conforming to Cho and Yoo study [47] internet usage amount did not show significant explanatory power for cyberbullying because the effect is related to the purpose of the use. Girls are more prone to use internet for social networking while boys are more users of video games, so they are less exposed to cyberbullying incidents compared to female students. Conforming to Barlet and Coyne [48] age can moderate the relationship. The authors found that girls were more likely to report more incidents during early stages and boys during later stages of adolescence.

Some scholars identified that the relationship between cybervictimization and loneliness is bidirectional [16,18,19,23]. Adolescents with high score of loneliness were found to be less skilled for face-to-face communication and, on the one hand, to be more open to socialize and create relationships in online settings but on the second hand to be more willing to disclose private information such as personal data, photos, home address, phone number, passwords, location, etc increasing the risk of victimization [49,50,51].

A good relationship with parents and a positive school climate were found to be protective factors [18,51]. Apart from traditional bullying, cyberbullying is transpassing the school environment. because aggression can occur anywhere and anytime. As studies showed, victims usually disclose the incidents to their peers and less to adults, but parents and teachers have an important role in identifying cyberbullying signs. Parents’ knowledge and awareness of cyberbullying signs have a significant role in the prevention of digital bullying [52]. Floros and Mylona [53] showed that security practices exercised by parents had a protective role in case of victimization but less in case of perpetration. Additionally, a survey conducted by Kowalski and his team [54] on American parents revealed that 93% of respondents believed that their children do generally good things through the Internet and 65% of them were confident that their children were at no risk while on the Internet. These results showed that the first-line guardians in case of victimizations are not always informed about the risks of online settings [55].

Our study has important implications in the school and family field because it provides insights to orient teachers’ interventions and parental education training that could improve relationships with adolescents. Both the school climate (stronger and more confident relationships with teachers and a satisfactory relationship with classmates) and a better relationship with their parents will assure a healthier psychological and social life.

### 4.1. Strengths and Limitations of the Study

The results of the present study are important, especially because the research on cyberaggression and cybervictimization among Romanian adolescents is not abundant. The characteristics considered for the research are important so the results can be generalizable (both female and male respondents; different years of study; different ages; enrolled in public and private schools; scientific or vocational specializations; and coming from different regions of the country). The results present cyberbullying and cybervictimization in relationship with parental style, FAS [56], and sociodemographic variables, and consider the role of cyberaggression and cyberbullied students. 

There are some limitations of the study. The research did not take into consideration psychological and psychiatric troubles, mental and physical deficiencies, or diagnostics of chronic diseases or behavioral-related problems. Previous studies have shown that there is an increased risk of cyberbullying for children with obesity, mental or physical diseases, or chronic illnesses. For example, it was found that children with visual impairment have an 80% higher risk of being victims of bullying [57,58]. Additionally, the study did not ask about their sexual orientation since some researchers showed that this variable could increase the risk of being bullied and cyberbullied. Therefore, considering the comparative results, the present study did not take into consideration cultural issues related to parenting styles that were proven by few researchers to be important when it comes to family rules and authoritarian parenting styles.

### 4.2. Reflections and Planning

In the case of cyberbullying, the approaches must transcend the school and family and must include community, parental education, health interventions, and other institutional stakeholders (policemen, counselors, psychotherapists, and lawyers) to intervene early, to prevent social marginalization and victimization, to explain the impact of cyberaggression and the social and legal consequences, and to act in cases of need. 

Findings from different studies indicated a vulnerability to cyberbullying during early adolescence suggesting that early, preventive, and context-appropriate interventions may be necessary to impact this phenomenon and diminish its consequences. Adolescence represents a tumultuous and vulnerable period of transformation, and sometimes the consequences of cyberbullying transcend to adulthood and could be life-threatening.

## 5. Conclusions

This research confirms the major role that parenting plays in their children’s involvement in bullying and cyberbullying, along with the school environment (relationships with classmates and teachers). Cyberaggression and cybervictimization were found to be related to several factors, and therefore, due to their diverse aspects (personal, familial, educational, social and psychological), the interventional programs and educational training must also be focused on adolescents, teachers and parents. The results of this study are important in order to construct appropriate approaches considering the great impact of cyberaggression and cybervictimization on the quality of life during adulthood.

## Figures and Tables

**Table 1:** Family-related data—parents’ level of education **^1^**.

Variables	M ± S.D and % *
Mothers’ educational level	
Primary school	9 (1.1%)
Secondary school	106 (12.7%)
High school	404 (48.4%)
University	245 (29.3%)
I do not know	71 (8.5%)
Fathers’ educational level	
Primary school	9 (1.1%)
Secondary school	98 (11.7%)
High school	426 (51.0%)
University	205 (24.6%)
I do not know	97 (11.6%)

* ^1^ Means and standard deviations (M ± D), frequency and percentages (%).

**Table 2:** Self-rated items regarding satisfaction with relationship with different categories of people ^1^.

On a Scale of 1 to 5, How Satisfied Are You with the Relationship with	1	2	3	4	5	M ± SD
your parents	21	53	150	203	408	4.10 ± 1.06
	(2.5)	(6.3)	(18.0)	(24.3)	(48.9)	
your friends	7	35	91	327	375	4.23 ± 0.86
	(0.8)	(4.2)	(10.9)	(39.2)	(44.9)	
your classmates	31	77	247	294	186	3.63 ± 1.04
	(3.7)	(9.2)	(29.6)	(35.2)	(22.3)	
other students in the school	85	435	263	33	19	2.36 ± 0.80
	(10.2)	(52.1)	(31.5)	(4.0)	(2.3)	
teachers	34	78	253	300	170	3.59 ± 1.03
	(4.1)	(9.3)	(30.3)	(35.9)	(20.4)	

^1^ Number of answers (N) and percentage (%), means and standard deviations (M ± SD).

**Table 3:** Self-rated items regarding agreement with different items ^1^.

Items: On a Scale of 1 to 5, I Consider That I Am A.	1	2	3	4	5	M ± SD
popular person	127	211	381	95	21	2.60 ± 0.96
	(15.2)	(25.3)	(45.6)	(11.4)	(2.5)	
solitaire person	62	99	303	314	57	3.24 ± 1.00
	(7.4)	(11.9)	(36.3)	(37.6)	(6.8)	
leader in my group	154	255	325	75	26	2.47 ± 0.99
	(18.4)	(30.5)	(38.9)	(9.0)	(3.1)	

^1^ Number of answers (N) and percentage (%), means and standard deviations (M ± SD).

**Table 4:** Reporting a cyberbullying behaviour ^1^.

Have You Ever Report to an Adult When You Saw a Kid Being Bullied Online (Messages, Social Media, Chatrooms, etc.)?	5.98 ± 2.62
Yes, to my parent	121 (14.5%)
Yes, to the kid’s parent	32 (3.8%)
Yes, to a teacher	61 (7.3%)
Yes, to the school psychologist	6 (0.7%)
Yes, to the principal	2 (0.2%)
To other adult	44 (5.3%)
No, I did not report any incident	218 (26.1%)
I did not report ay incident because I did not see any	351 (42.0%)

^1^ Means and standard deviations (M ± D), frequency and percentages (%).

**Table 5:** Correlational results of CYB-AGS and CYVIC subscales.

Items	ICYB-AGS	DCYB-AGS	Impersonation	Visual- Sexual	Written Verbal	Online Exclusion
Have you ever been bullied online?	r = 0.405 **	r = 0.307 *,	r = 0.138 **	r = 0.241 **	r = 0.284 **	r = 0.141 **
	*p* = 0.000	*p* = 0.000	*p* = 0.000	*p* = 0.000	*p* = 0.000	*p* = 0.000
Have you ever bullied others while online?	r = 0.373 **	r = 0.304 **	r = 0.089 **	r = 0.180 **	r = 0.227 **	r = 0.138 **
	*p* = 0.000	*p* = 0.000	*p* = 0.010	*p* = 0.000	*p* = 0.000	*p* = 0.000
UCLA scale	r = 0.170 **	r = 0.180 **	r = 0.185 **	r = 0.234 **	r = 0.294 **	r = 0.453 **
	*p* = 0.000	*p* = 0.000	*p* = 0.000	*p* = 0.000	*p* = 0.000	*p* =0.000
PSDQ scale	no	no	no	no	r = −0.069 *	r = −0.102 **
	correlation	correlation	correlation	correlation	*p* = 0.046	*p* = 0.003

* *p* < 0.05; ** *p* < 0.001.

**Table 6:** Correlational results of PSDQ subscales and aggression and victimization scores *.

Items	Supportive Parents	Controlling Parents	Compassionate Parents	Aggressive Parents	Avoidant Parents	Orthodox Parents
CYB-AGS score	no	no	r = −0.082 *	r = 0.170 *,	r = −0.168 **	no
	correlation	correlation	*p* = 0.017	*p* = 0.000	*p* = 0.000	correlation
CYVIC scores	r = −0.095 **	no	r = −0.151 **	r = 0.172 **	r = −0.230 **	no
	*p* = 0.006	correlation	*p* = 0.000	*p* = 0.000	*p* = 0.000	correlation

* *p* < 0.05; ** *p* < 0.001.

## Data Availability

Data availability upon request on corresponding author.
